# RNA-seq analysis of antibacterial mechanism of *Cinnamomum camphora* essential oil against *Escherichia coli*

**DOI:** 10.7717/peerj.11081

**Published:** 2021-03-17

**Authors:** Yutian Yu, Jie Dong, Yanlu Wang, Xi Gong

**Affiliations:** Human Aging Research Institute and School of Life Science and Jiangxi Key Laboratory of Human Aging, Nanchang University, Nanchang, Jiangxi, China

**Keywords:** *Escherichia coli*, * Cinnamomum camphora* essential oil, Antibacterial mechanism, RNA-seq, Gene expression regulation

## Abstract

**Background:**

Transcriptome analysis plays a central role in elucidating the complexity of gene expression regulation in *Escherichia coli*. In recent years, the overuse of antibiotics has led to an increase in antimicrobial resistance, which greatly reduces the efficacy of antibacterial drugs and affects people’s health. Therefore, several researchers are focused on finding other materials, which could replace or supplement antibiotic treatment.

**Methods:**

*E. coli* was treated with water, acetone and *Cinnamomum camphora* essential oils, respectively. The antibacterial activity was assessed using the minimum inhibitory concentration (MIC), the minimum bactericidal concentration (MBC), the dry weight and the wet weight of the cells. To explore the antibacterial mechanism of the oil, the RNA-Seq analysis was adopted under three different treatments. Finally, the expression of related genes was verified by Quantitative PCR.

**Results:**

In this study, we showed that the* C. Camphora* essential oil exerted a strong antibacterial effect. Our results showed that the inhibitory efficiency increased with increasing of the concentration of essential oil. RNA-seq analysis indicated that the essential oil inhibited the growth of *E. coli* by inhibiting the metabolism, chemotaxis, and adhesion, meanwhile, life activities were maintained by enhancing *E. coli* resistance reactions. These results are contributed to uncover the antimicrobial mechanisms of essential oils against *E. coli*, and the *C. Camphora* essential oil could be applied as an antibacterial agent to replace or ally with antibiotic.

## Introduction

*Escherichia coli*, discovered in 1884 by a German biologist, is classified as a rod-shaped, Gram-negative bacterium in the family Enterobacteriaceae. The bacterium mainly inhabits the lower intestinal tract of warm-blooded animals, including humans, and is often discharged into the environment through faeces or wastewater effluent. Many gene manipulation systems have been developed using *E. coli* as the host bacterium, producing countless enzymes and other industrial products. Genome sequence analysis of *E. coli* was first reported in 1997. Since then, more than 4800 *E. coli* genomes have been sequenced (Jang et al., 2017). *E. coli*, paradoxically, is also one of the main pathogens, being responsible for both intraintestinal and extraintestinal infections. In some cases, some *E. coli* strains multiply and become pathogenic pathogens, and cause diseases such as diarrhea, peritonitis, colitis, bacteremia, urinary tract infections, and in severe cases, kidney failure and cancer (Welch et al., 2002; Kaper, 2005; Arthur et al., 2012; Russo & Johnson, 2003).

In the past few decades, the extensive use of antibiotics led to the development of drug-resistant strains, which especially contributed to emergent antibiotic-resistant pathogens and potentially resistant organisms (Davies, 2007; Davies, 1996). Considering, several researchers are focused on finding other materials, which could replace or supplement antibiotic treatment natural green essential oils may be the best choices (Bakkali et al., 2008).

Essential oils, which are extracted from all parts of plants, are volatile oils. To date, several studies documented that plant essential oils possess the efficacy of inhibiting pathogens: e.g., *Ocimum basilicum* essential oil inhibits *Cryptococcus* growth (Cardoso et al., 2017), *Clove* essential oil inhibits *Campylobacter jejuni* (Kovács et al., 2016), and *Perilla* oil inhibits *Staphylococcus aureus* (Qiu et al., 2011). In addition, when combined with antibiotics, they exhibit to inhibit the growth of drug-resistant strains (Langeveld, Veldhuizen & Burt, 2014). The essential oil was applied to clinical treatment; for example, as a stimulant, it has been administered internally to treat mild muscle pain, muscle congestion, breast pain; but as an analgesic and antipruritic agent, when applied to external treatment (Van Wyk, Van Oudtshoorn & Gericke, 2009). Essential oils can even be adopted to treat cancer (Sylvestre et al., 2006).

*Cinnamomum camphora* (*C. Camphora*) essential oil has a variety of biological properties, and its application as an antibacterial agent is increasing gradually. Numerous reports had implicated that *C. camphora* essential oil not only exerts a strong antibacterial effect on *Candida albicans*, *Saccharomyces cerevisiae*, and other gram-positive bacteria, but also exerts an inhibitory effect on gram-negative bacteria such as *E. coli* and *S. aureus* (Juteau et al., 2002; Santoyo et al., 2005). Furthermore, the vapor-phase of *C. camphora* essential oils possessed significant antibacterial activity (Wu et al., 2019). In addition, *C. camphora* essential oil also was implicated in the effect on antiviral, anti-cough (Tirillini, Velasquez, and Pellegrino 1996; Kamdem & Gage, 1995; Viljoen et al., 2003; Hammerschmidt et al., 1993).

Currently, the bacteriostatic mechanism associated with *E. coli* only has been partially illustrated. For *C. camphor* essential oil, the bacteriostatic mechanism associated with *E. coli* has never been reported. In this study, *E. coli* was treated with water, acetone, and *C. camphora* essential oils, respectively, and then the morphology of *E. coli* was analyzed. Then, we explored the inhibitory mechanism of *C. camphora* essential oil against *E. coli* by using RNA-seq techniques. The transcriptomic data showed that essential oil inhibited the metabolism, chemotaxis, and some genes related to the resistance reactions of *E. coli*. These findings are helpful to expand the understanding of the antimicrobial mechanisms of essential oils against *E. coli*.

## Materials & Methods

### Materials

The 98% *C. camphora* essential oil used in this study was purchased from Chengdu Aikeda Chemical Reagent Co. Ltd and the acetone was from Xilong Scientific Co. Ltd. *E. coli* ATCC8739, provided by the Strain Preservation Center, was cultured in Luria-Bertani (1% sodium chloride, 1% peptone, and 0.5% yeast extract).

### Evaluation of antibacterial activity

#### Assay of antibacterial activity

The LB solid medium was poured into the culture dish, and the solidified medium covered only the bottom. After putting into four Oxford cups, appropriate LB solid medium was added. After medium solidified, took out the Oxford cup and evenly spread the cultured *E. coli* on the solid medium, carefully avoid the four wells. Then, equal amounts of agent, essential oil, ampicillin, acetone, and water, were placed into four wells, respectively. Cultured at 37 °C, overnight.

#### Analysis of the growth of *E. coli*

A total of 1 ml of agent, which is water, acetone, and *C. camphor* essential oil respectively, was injected into a 3 ml LB liquid medium, and three replicates for each group. The *E. coli* was inoculated at 1:100, and incubated at 37 °C and 200 rpm. After shaking culture for 24 h, the bacteria were collected by centrifugation and the wet weight was weighed. In order to obtain dry weight, the bacteria were put into the drying closet (60 °C) and weighed several times until the constant weight was obtained (Fan et al. 2015). The wet weight and dry weight are the average of three repeated experiments. Dry and wet weights should be measured at a fixed time each day for 7 consecutive days.

### Determination of MIC and MBC

Minimum inhibitory concentration (MIC) determination is to determine the lowest concentration of the essential oil that inhibits the growth of bacteria. MIC was defined as the concentration for the transition from a plate full of colonies to a dense single colony. Minimum bactericidal concentration (MBC) determination is to determine the minimum concentration of the essential oil that kills bacteria, which was defined the minimum concentration for no bacterial colony growth as the MBC. After treating *E. coli* with acetone (100% concentration) and water, the wet and dry weights of them were measured to determine the inhibitory effect of the essential oil on the growth of them. After different concentrations of acetone and the essential oil were applied to *E. coli*, the inhibitory effects of the different concentrations of acetone on the growth of *E. coli* were determined according to the growth of the plate colony and turbidity of the culture medium during culture. All the experiments were repeated in triplicate.

### Effects of the essential oil on the cell membrane and cell wall of *E. coli*

With water as a control, *E. coli* ATCC8739 was treated with acetone and 1/2 MIC of the essential oil to determine the cell integrity and effect of the essential oil on the cell permeability. In order to detect β-galactosidase (β-Gal) and alkaline phosphatase (AKP) leakage in the supernatant, the β-galactosidase enzyme activity and alkaline phosphatase assay kits (Solarbio) were used respectively.

### RNA-seq analysis

*E. coli* was first treated with lysozyme for 5 min at room temperature. Then, total RNA was extracted using RNAiso (TakaRa, D9108A) according to the manufacturer’s instructions. Then the biomass was stored at −80 ^∘^C until further treatment. RNA purity was assessed using a NanoDrop ND-1000 Spectrophotometer (Isogen Life Science) and 1% agarose gel electrophoresis. Then the high-quality RNA was sent to the Beijing Novogene Bioinformatics Technology Co., Ltd (Beijing, China).

In order to obtain the high-quality clean data, the raw reads from the Beijing Novogene Bioinformatics Technology Co., Ltd were initially processed for removing the adapter sequences and low-quality reads. Clean Reads were quickly and accurately compared with the reference genome of *E. coli* by using Bowtie2 to obtain the localization information of Reads on the reference genome. After the quantification of gene expression was completed, DESeq2 was used to conduct statistical analysis on the expression data, and the genes with significantly different expression levels in different states were screened. GOseq was used in Gene Ontology (GO) enrichment analysis which was based on Wallenius non-central hyper-geometric distribution. KOBAS (2.0) was used for Pathway significant enrichment analysis, which took pathways in the Kyoto Encyclopedia of Genes and Genomes (KEGG) database as units, and applied hypergeometric test to find the pathways that showed significant enrichment in differentially expressed genes compared with the whole genome background.

### Real-time polymerase chain reaction

Total RNA was reverse transcribed into cDNA using the PrimeScript RT reagent kit (Takara, Japan). Real-time polymerase chain reaction (RT-PCR) was performed using the SYBR Green Premix Ex Taq II (Takara, Japan) and Applied Biosystems StepOnePlus real-time PCR System (Applied Biosystems, Carlsbad, CA, USA).

## Results

### Antibacterial activity

**Figure 1:**
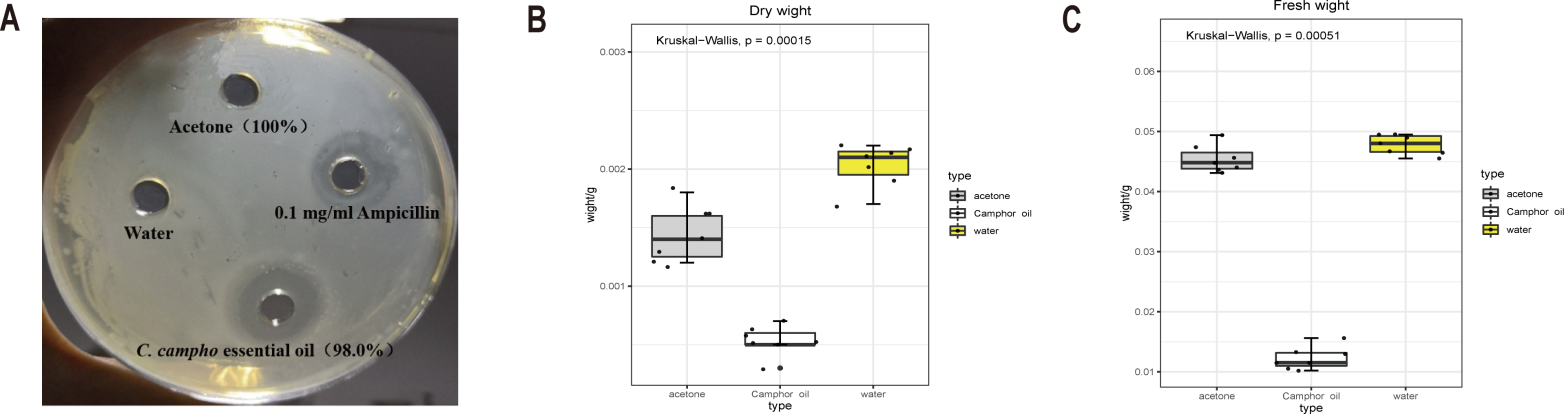
The essential oil and ampicillin solution show stronger bacteriostatic activity. The dry and fresh weight of *E. coli* was significantly reduced after treatment with the essential oil. (A) Diameter of bacteriostatic circle for four treatments. (B) Fresh weight and (C) dry weight of *E. coli* after treatment with C. camphora essential oil (100% concentration), acetone (100% concentration) and water.

To investigate the bacteriostatic activity, the Oxford cup method was used to detect the bacteriostatic circle. Compared with water and solvent acetone, which have no bacteriostatic phenomenon, the essential oil and ampicillin solution show stronger bacteriostatic activity (Fig. 1A). The antibacterial activity of ampicillin solution was slightly higher than that of the essential oil, but there was no significant difference, and the inhibitory zone size was between 11-11.5 mm (Table 1). This result suggested that the essential oil (100% concentration) had antibacterial effect on *E. coli*, and had similar antibacterial activity as 0.1 mg/ml ampicillin solution.

**Table 1:** Diameter of bacteriostatic circle for four treatments (mm).

	Water	Acetone	Camphor oil	0.1 mg/ml ampicillin
Diameter	0	0	11 ± 0.5	11.5 ± 0.6

Comparing the control and acetone, the dry and wet weights of *E. coli* reduced significantly after treatment with the essential oil (Figs. 1B, 1C). The dry and wet weights of *E. coli* treated with acetone, the solvent of the essential oil, were also less than those treated with water. This indicated that the essential oil exerted a strong antibacterial effect on *E. coli*, and acetone also had certain inhibitory effect on the growth of *E. coli*. Therefore, different concentrations of acetone were applied to *E. coli*, and the inhibition effect of the different concentrations of acetone on the growth of *E. coli* was determined according to the growth of the plate colony and turbidity of the culture medium during culture. (Table 2). The results showed that acetone did not exert a strong bacteriostatic effect. To ensure the minimum effect of the solvent on the bacteriostatic phenomenon and at the same time to dissolve the essential oil, 5% acetone concentration was selected as the concentration of dissolved the essential oil.

**Table 2:** Inhibition effect of acetone at different concentrations.

Concentration Percentage (%)	Colony growth	Turbid degree
2.5–5	Complete coverage	Turbidity
7.5–10	Complete coverage	Slight turbidity
12.5–15	Dense colonies	Clear
17.5–25	Sparse colonies	Clear

The MIC and MBC of the essential oil are shown in Table 3. This indicated that the essential oil exerted a strong bacteriostatic effect on *E. coli*, and the MIC value was 0.625%. The MBC of the oil against *E. coli* was 2.5%. Therefore, to explore the antibacterial mechanism of the essential oil on *E. coli*, the 1/2 MIC of the essential oil concentration was selected to treat *E. coli.* In this case, the *E. coli* plate colony showed growth, but the growth rate was much lower than that of the control.

**Table 3:** Inhibitory effect of camphor oil with different concentration.

Concentration Percentage (%)	Colony growth	Turbid degree
2.5	No colony	Clear
1.25	Sparse colonies	Clear
0.625	Dense colonies	Clear
0.3125	Complete coverage	Slight turbidity

### Effects of the subinhibitory concentration of the essential oil on the cell membrane and cell wall of *E. coli*

**Figure 2:**
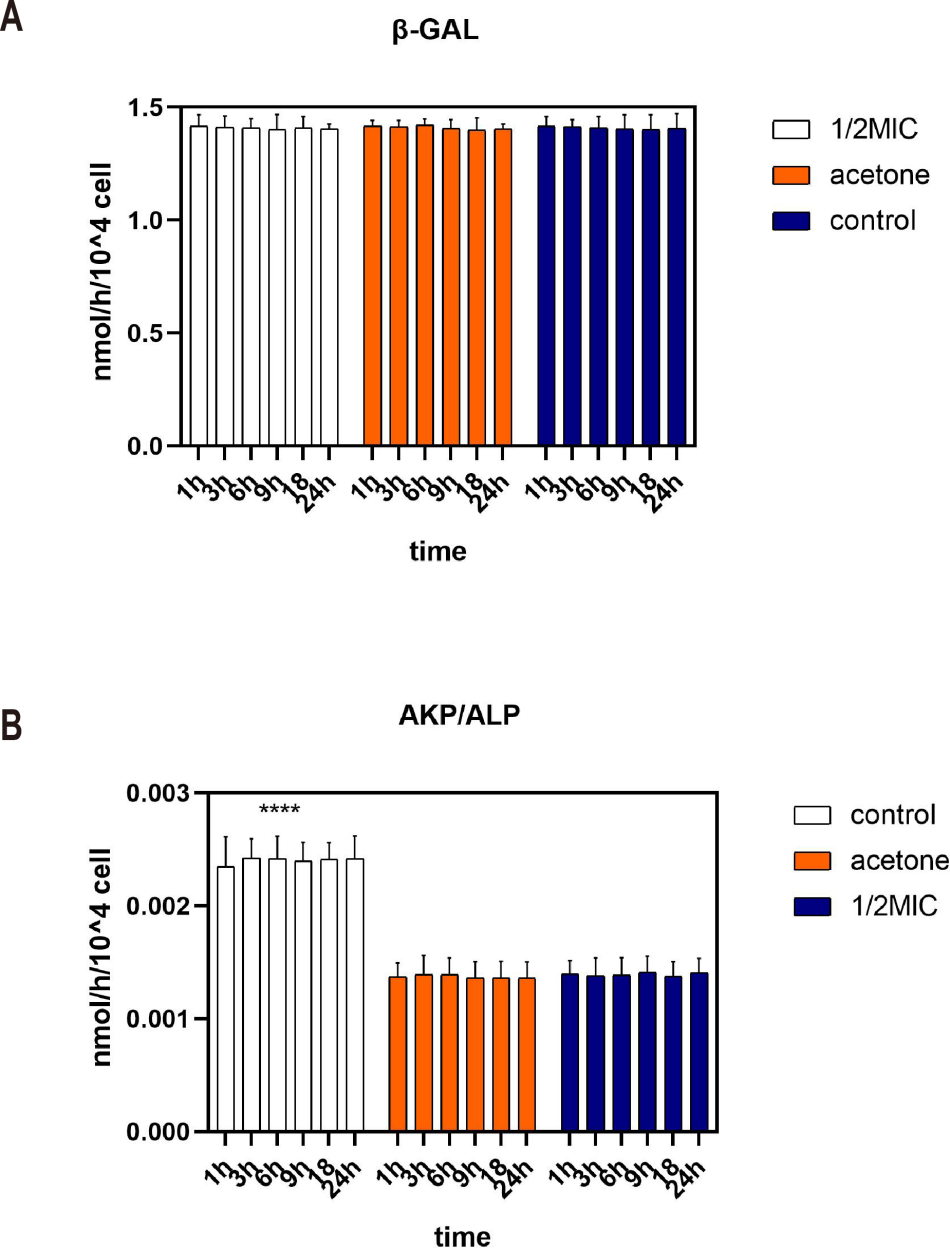
Effects of *C. camphora* essential oil on cell membrane (A) and cell wall (B) integrity of *E. coli*.

Beta-galactosidase is an intracellular enzyme which could not be detected in supernatant. Figure 2A shows that the content of β-galactosidase enzyme in the supernatant was very low during the 24 h analysis. The consistency occurs in three treatments. This indicated that 1/2 MIC of the essential oil and acetone did not inhibit the growth of *E. coli* by destroying the cell membranes.

Alkaline phosphatase (AKP) is a protease that exists between cell membranes and cell walls. It is almost impossible for AKP to pass through the cell wall of a healthy bacterium. Moreover, AKP activity was higher in the control group than in the acetone and the essential oil (Fig. 2B). Therefore, acetone and 1/2 MIC of the essential oil did not destroy the cell wall of *E. coli* and even prevented the cell wall from being destroyed.

### Identification of differentially expressed genes

The difference in expression is determined by understanding the difference genes between different treatment to clarify the gene regulation. Then the DEseq package and setting the parameter padj at < 0.05 were adopted to identify the differentially expressed genes.

**Figure 3:**
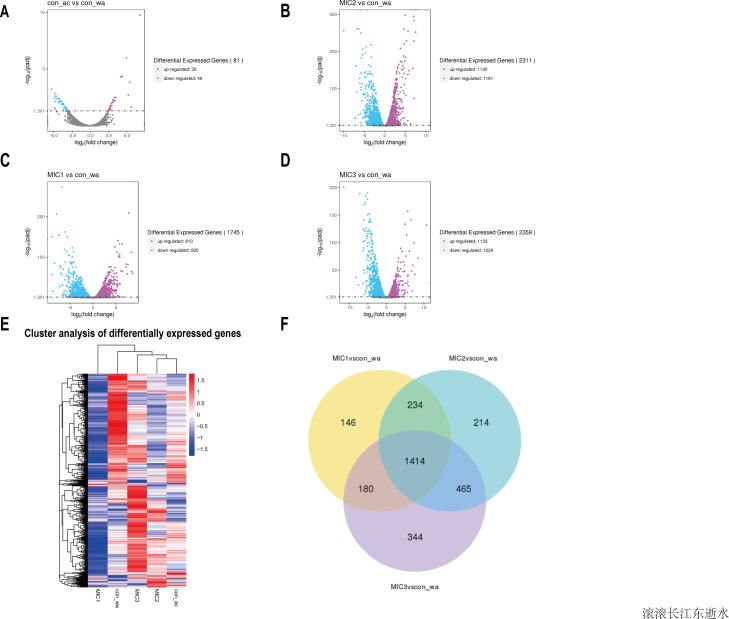
Overview of the gene expression analysis. (A–D) In comparison with water treatment, the volcano diagrams of differential genes after acetone and different concentrations of *C. camphor* essential oil treatment. (The horizontal axis shows the fold change of genes in different samples. The vertical axis shows the statistically significant degree of changes in gene expression levels. The points represent genes. Gray dots indicate no significant difference in genes, purple dots indicate upregulated differential expression genes, blue dots indicate downregulated differential expression genes). Hierarchical clustering heat map of different gene expressions in different experimental conditions. Red represents high gene expression and blue represents low gene expression. (F) Differential gene Venn diagram. (With water treatment as the control, differential gene Venn diagram of different concentrations of the essential oil treated.

To investigate the effect of the essential oil on the gene expression of *E. coli*, upregulated and downregulated genes were assessed (Figs. 3A–3D). The results showed that there were only 81 different genes in the acetone-treated compared with the control, consistent with previous results. Nevertheless, there were 1745, 2311, and 2359 differential genes in the 1/8 MIC, 1/4 MIC, and 1/2 MIC of the essential oil-treated compared with the control, respectively. For the concentration of essential oil, the number of the different genes were increased. Among them, the downregulated genes increased with an increase in concentration, but the upregulated genes increased first and then stabilized. There was little difference in the upregulated genes, compared with the 1/4 MIC and 1/2 MIC of the essential oil. This result indicated that the concentration of 1/4 MIC of the essential oil was an equilibrium point. Perhaps at this concentration, the regulation of growth in *E. coli* reached its maximum value.

To analyze gene expression patterns of differentially expressed genes in different processing states, cluster analysis was adopted. Genes with similar expression patterns may have similar functions, or participate in the same metabolic process, or participate in the same cell pathway. Therefore, these genes with similar expression patterns were grouped into classes to examine the changes in gene expression under different treatment conditions and the gene expression changes were examined after gene normalization. The results showed that the gene expression patterns were different among the different treatments, indicating that the metabolic pathways were different among the different treatments (Fig. 3E).

To understand how the genes were classified, a Venn diagram of the different genes was constructed. The results showed 1,414 identical differentially expressed genes in *E. coli* treated with three concentrations of the essential oil compared with the control (Fig. 3F). The function of these common differentially expressed genes may be related to *E. coli* resistance.

### GO analysis of the differential genes

**Figure 4:**
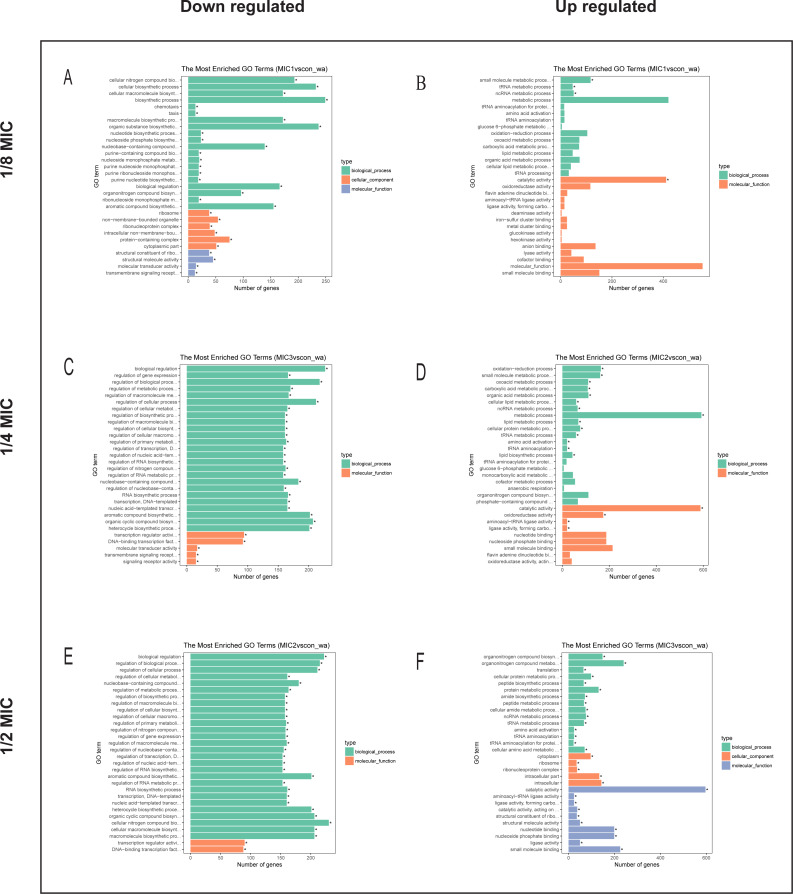
The difference gene of *E. coli* after action of (A, B) 1/8 MIC, (C, D) 1/4 MIC and (E, F) 1/2 MIC *C. camphor* essential oil GO term.

To explore the antibacterial mechanism of the essential oil, GO term enrichment analysis was performed on the differential genes of treatment with 1/8, 1/4, and 1/2 MIC of the essential oil (Fig. 4). The differential genes under different treatments were introduced into the R Programming; the GOseq package was employed to enrichment analysis and the hmmscan package was used for annotation. The 30 GO terms with the most significant enrichment were selected and plotted in the graph. In addition, to understand the GO terms which were significantly enriched in the GO analysis, a directed acyclic graph (DAG) analysis was performed to draw the DAG diagrams of the biological process (BP), molecular function (MF), and cellular component (CC). Data files were given as Figs. S1–S3. The non-analytic and chaotic nodes were screened out and analytic and significantly enriched nodes were sorted out for viewing by using Visio. In Figs. S1–S3, each ellipse node represents a GO term, and the box represents the GO with an enrichment degree of TOP 10. For the 1/8 MIC, the downregulated pathways were enriched in the process of bacterial chemotaxis, anabolic metabolism, signal transduction, etc., while the upregulated pathways were mostly enriched in productive metabolic activities and resistance activities. For the 1/4 MIC, the downregulated pathways were enriched in the regulation of gene expression, the process of macromolecule anabolic metabolism, and signal transduction, while the upregulated pathways were enriched in the production capacity reaction and the synthesis of some resistant substances. For the 1/2 MIC, the downregulated pathways were enriched in the synthesis, signal transduction, and metabolism processes of macromolecules, while the upregulated pathways were enriched in the synthesis and translation processes of some resistant substances.

### KEGG pathway analysis of differentially expressed genes

**Figure 5:**
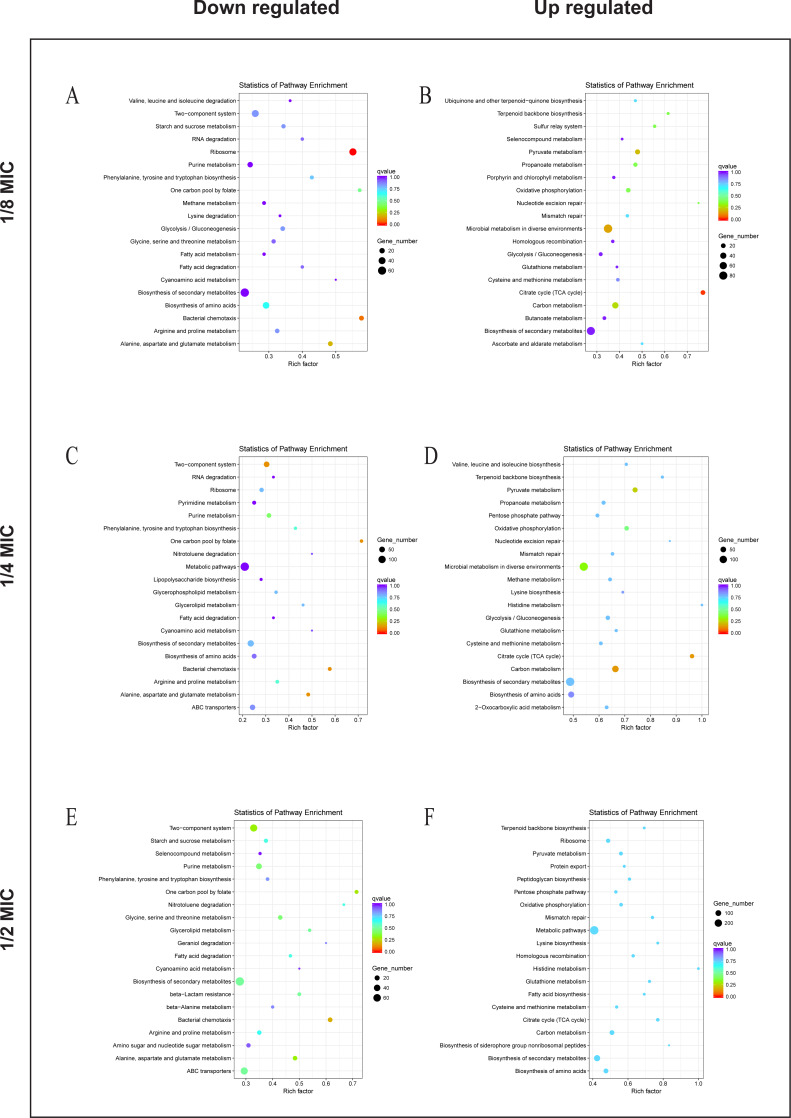
KEGG Pathway of *E. coli* differential genes. KEGG Pathway of *E. coli* differential genes at (A, B) 1/8 MIC, (C, D) 1/4 MIC, and (E, F) 1/2 MIC.

To explore the antibacterial mechanism of the essential oil, KEGG pathway enrichment analysis (Fig. 5). According to KEGG pathway analysis, the growth of *E. coli* was inhibited through inhibiting the anabolic, chemotaxis, signal transduction, and other life activities of *E. coli*, while the activity was maintained by strengthening its production capacity and repair in the 1/8 MIC of the essential oil. In the 1/4 MIC of the essential oil, the growth of *E. coli* was inhibited by inhibiting metabolism, movement, signal transduction, and other life activities as well as some resistance activities, while life activities were maintained by increasing production capacity and enhancing the synthesis of resistant substances. In the 1/2 MIC of the essential oil, the growth of *E. coli* was inhibited by inhibiting metabolism, chemotaxis, and certain resistance reactions, while life activities were sustained by enhancing productive reaction, the translation process, and the synthesis of resistant substances. These results indicated that the essential oil exerted a strong antibacterial effect against *E. coli*, and this inhibitory effect was realized by inhibiting the life activity, signal transduction and inhibitory activity and enhanced with an increase in concentration.

### Analysis of the bacteriostatic mechanism

**Figure 6:**
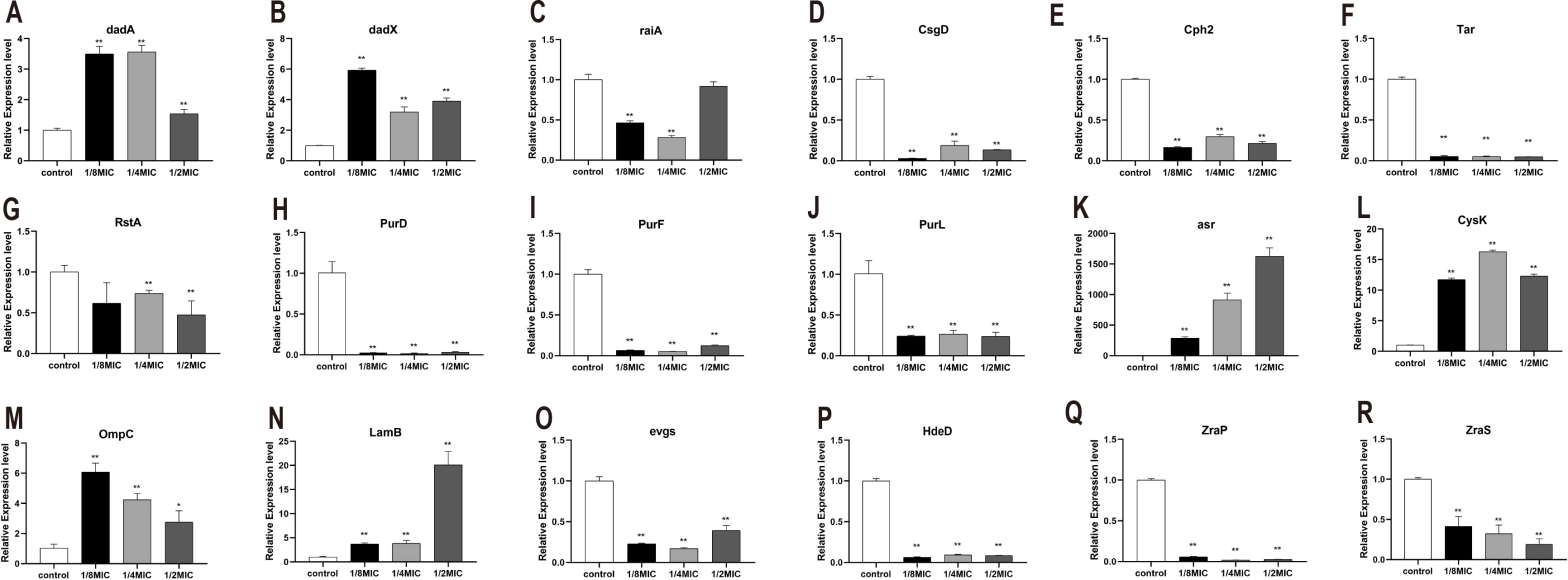
(A-R) The effects of *C. camphor* oil on chemotaxis, metabolism, productivity, cell structure, resistance related genes, antibiotic sensitivity and resistance related gene expression in *E. coli* were determined by polymerase chain reaction. n = 3 for each group.

To explore the bacteriostatic mechanism, several genes which encode metabolism and resistance in *E. coli* were selected and measured by RT-PCR. Eighteen functionally relevant genes (*dadA*, *raiA*, *dadX*, *csgD*, *tar*, *cph2*, *rstA*, *purD*, *purF*, *purL*, *hdeD*, *evgs*, *zraS*, *zraP*, *asr*, *cysK*, *ompC*, and *lamB*) were randomly selected to evaluate the transcription levels. For quantitative real-time PCR, the results showed that the *dadA*, *dadX*, *asr*, *cysK*, *ompC*, and *lamB* expression levels were significantly higher in treated with essential oil than the control (Fig. 6). Nevertheless, the expression of *raiA*, *csgD*, *cph2*, *tar*, *rstA*, *purD*, *purF*, *purL*, *hdeD*, *evgs*, *zraS*, and *zraP* were significantly inhibited (Fig. 6).

## Discussion

*Escherichia coli* is a Gram-negative bacterium that generally acts as a natural commensal in the digestive tracts of humans and animals, but some strains are significant intestinal and extraintestinal pathogens which can cause a variety of diseases, ranging from self-limiting gastrointestinal infections to bacteremia. In this study, we showed that the *C. Camphora* essential oil exerted a strong antibacterial effect. To explore the antibacterial mechanism of the essential oil on *E. coli*, the 1/2 MIC was selected to treat *E. coli*. Previous studies have shown that allyl isothiocyanate (AITC), a natural compound present in the family Cruciferae, has been demonstrated to have a strong antimicrobial activity through damaging cell membranes and causing leakage of cellular metabolites (Lin, Preston & Wei, 2000). Cell membrane integrity plays an important role in cell growth. Since β-galactosidase exists in the cytoplasm, and alkaline phosphatase is located between the cell wall and the cell membrane, the increase in enzymes activity of the culture medium is a measure of the extent to which cells are rendered permeable or lysed because of the test chemicals on the cell membrane (Lin, Preston & Wei 2000; Nowicki et al., 2016). Our results were suggested that the antibacterial action of *C. Camphora* essential oil is not achieved by impairment of bacterial membrane integrity.

According to our RNA-Seq analysis, the antimicrobial action of *C. Camphora* essential oil was suggested to be related to disruption of major function in inhibiting the life activity, signal transduction and inhibitory activity. Subsequently, eighteen functionally relevant genes were randomly selected to evaluate the transcription levels.

D -Alanine is a central component of the cell wall in most prokaryotes. In bacterial, *dadA* encodes the D -alanine dehydrogenase (DadA) that catabolizes d-alanine to pyruvate and ammonia. Trivedi *et al.* utilized a high-throughput methodology for screening cell mechanics to discover that deletion of *dadA*, leads to a 3-fold reduction in the bending rigidity of *P. aeruginosa* cells. In a *dadA* loss-of-function mutant, higher intracellular levels of D-alanine inhibit expression of *ponA* and *dacC*, which encode cell wall enzymes, and lead to a decrease in cell wall cross-linking (Odermatt et al., 2018; Trivedi et al., 2018). In *E. coli*, the *dadX*, which encodes alanine racemase, is essential for L-alanine catabolism, and provides a secondary source of D-alanine for cell wall biosynthesis (Kang et al., 2011; Wild et al., 1985). Our results showed that the expression of *dadA* and *dadX* were significantly upregulated after treated with the essential oil, consistent with the experimental about the integrity of the cell membrane and cell wall. Our results indicated that *C. Camphora* essential oil does not impair to cell membrane and wall and even seems to protect them to a certain extent. Thus, we speculated that the essential oil could regulate the growth of *E. coli* by inhibiting the expression of ribosome-related genes, *raiA* gene is a ribosomal stable protein that maintains ribosomal stability (Tikunova et al., 2007; Zhukov et al., 2007), and its expression was significantly inhibited.

To explore the expressed difference of chemotaxis- and adhesion-related genes, several genes were detected. *CsgD*, the master regulator of biofilm formation, activates the synthesis of curli fimbriae and extracellular polysaccharides in *E. coli* (Ogasawara, Yamamoto & Ishihama, 2011). *Cph2* protein is a membrane-associated transcription factor that is processed to release the N-terminal DNA binding domain, is found to regulate hyphal development in a medium-specific manner (Lane et al., 2001; Lane et al., 2015). *RstA*, one of two-component signal transduction systems (TCSs) exists in bacterial, has been implicated in the regulation of bacterial virulence in *Vibrio alginolyticus*, *Salmonella typhimurium Photobacterium damselae Clostridioides difficile*, and avian pathogenic *E. coli* (Liu et al., 2019). In addition, *rstA* and *rstB* are also reported to critical regulators of adhesion, biofilm production, motility in bacteria (Huang et al., 2018). The expression of *csgD*, *cph2* and *rstA* were significantly inhibited which indicates that the essential oil could affect the adhesion and biofilm formation in *E. coli*. Chemotaxis, the movement of an organism toward or away from chemicals, is an important adaptive behavior of motile prokaryotes, such as *E. coli*. *Tar* encodes methyl-accepting chemotaxis protein that is a chemoreceptor which senses aspartate and exists as a functional homodimer (Goldberg et al., 2009). The expression of *tar* was significantly inhibited which indicates that the essential oil could inhibit the chemotaxis in *E. coli*.

Energy metabolism is a type of bacterial growth expression condition. When bacteria have strong vitality, their metabolism will be vigorous; otherwise, their metabolism will be restricted. Therefore, the expression of metabolism-related genes is an important indicator of the viability of *E. coli*. Our results shown that the oil significantly inhibited to express of *purD*, *purF* and *purL*. *PurD*, *purF* and *purL* are involved in nucleotide synthesis from phosphate ribose pyrophosphate (PRPP) to hypoxanthine nucleotide (Zhao et al., 2016). This indicates that the essential oil could inhibit the expression of metabolism-related genes which result in growth inhibition in *E. coli*.

Bacteria can survive under many harsh conditions through improving their resistance. Resistance genes will be highly expressed in response to the stressful or severe environment (Šeputiene et al., 2003). Such as, *asr* plays a role in survival under acid conditions (Šeputiene et al., 2004). Cysteine synthase A encoded by cysK catalyzes the synthesis of cysteine from O-acetylserine. Expression of cysK in *E. coli* is upregulated under lithium condition (Yamamoto et al., 2011). Besides, *OmpC* and *LamB* are regard as outer membrane proteins (OMP) in *E. coli*, which has been proposed that they are required under some harsh conditions in Gram-negative bacteria (Ozkanca et al., 2002; Lin et al., 2014). It was observed that *asr*, *cysK*, *OmpC*, and *LamB* were significantly upregulated under the essential oil. The resistance of *E. coli* is mainly attributed to the expression of resistance genes and its efflux system. It was observed that *E. coli* responds to the essential oil by increasing the expression of its own resistance genes in this study.

Moreover, some of antibiotic resistance genes, such as *hdeD*, *evgs*, *zraS* and *zraP*, which expresses to resistant to antibiotics, are not only harmful, but also have a great impact on human health (Mates, Sayed & Foster, 2007; Kato et al., 2000; Petit-Härtlein et al., 2015). Our results showed that these genes were significant inhibited after treated with the essential oil. This suggests that the *C. Camphora* essential oil will be a potential antibacterial agent, which could be applied as an antibacterial agent to replace or ally with antibiotic to solve the problem of bacterial resistance.

## Conclusions

In summary, the studies showed that the *C. camphora* essential oil exerted a strong bacteriostatic effect, and the inhibitory efficiency increased with the increase of the concentration of essential oil. The essential oil does not damage the cell membrane and wall of *E. coli* and even protects the cell wall from being damaged to a certain extent. To investigate the effect of the the gene expression after the treatment of *C. camphora* essential oil on *E. coli*, RNA-Seq was adopted. Our studies indicated that the essential oil inhibited the growth of *E. coli* by inhibiting the metabolism, chemotaxis, and adhesion. Our results are contributed to uncover the antimicrobial mechanisms of essential oils against *E. coli*, and the *C. camphora* essential oil could be applied as an antibacterial agent to replace or ally with antibiotic.

##  Supplemental Information

10.7717/peerj.11081/supp-1 Supplemental Information 1 DAG analysis of GO enrichment in *Escherichia coli* after 1/8MIC treatment. The DAG of BP in the down-regulated gene GO term The DAG of BP in the down-regulated gene GO term.

10.7717/peerj.11081/supp-2 Supplemental Information 2 DAG analysis of GO enrichment in *Escherichia coli* after 1/8MIC treatment The DAG of CC in the down-regulated gene GO term.

10.7717/peerj.11081/supp-3 Supplemental Information 3 DAG analysis of GO enrichment in *Escherichia coli* after 1/8MIC treatment The DAG of MF in the down-regulated gene GO term.

10.7717/peerj.11081/supp-4 Supplemental Information 4 DAG analysis of GO enrichment in *Escherichia coli* after 1/8MIC treatment The DAG of BP in the up-regulated gene GO term.

10.7717/peerj.11081/supp-5 Supplemental Information 5 DAG analysis of GO enrichment in *Escherichia coli* after 1/8MIC treatment The DAG of MF in the up-regulated gene GO term.

10.7717/peerj.11081/supp-6 Supplemental Information 6 DAG analysis of GO enrichment in *Escherichia coli* after 1/4MIC treatment The DAG of BP in the down-regulated gene GO term.

10.7717/peerj.11081/supp-7 Supplemental Information 7 DAG analysis of GO enrichment in *Escherichia coli* after 1/4MIC treatment The DAG of MF in the down-regulated gene GO term.

10.7717/peerj.11081/supp-8 Supplemental Information 8 DAG analysis of GO enrichment in *Escherichia coli* after 1/4MIC treatment The DAG of BP in the up-regulated gene GO term.

10.7717/peerj.11081/supp-9 Supplemental Information 9 DAG analysis of GO enrichment in *Escherichia coli* after 1/4MIC treatment The DAG of MF in the up-regulated gene GO term.

10.7717/peerj.11081/supp-10 Supplemental Information 10 DAG analysis of GO enrichment in *Escherichia coli* after 1/2MIC treatment The DAG of BP in the down-regulated gene GO term.

10.7717/peerj.11081/supp-11 Supplemental Information 11 DAG analysis of GO enrichment in *Escherichia coli* after 1/2MIC treatment The DAG of MF in the down-regulated gene GO term.

10.7717/peerj.11081/supp-12 Supplemental Information 12 DAG analysis of GO enrichment in *Escherichia coli* after 1/2MIC treatment The DAG of BP in the up-regulated gene GO term.

10.7717/peerj.11081/supp-13 Supplemental Information 13 DAG analysis of GO enrichment in *Escherichia coli* after 1/2MIC treatment The DAG of CC in the up-regulated gene GO term.

10.7717/peerj.11081/supp-14 Supplemental Information 14 DAG analysis of GO enrichment in *Escherichia coli* after 1/2MIC treatment The DAG of MF in the up-regulated gene GO term.

10.7717/peerj.11081/supp-15 Supplemental Information 15 Wet weight and dry weight of *E. coli* after treatment with *C. camphor* essential oil (100% concentration), acetone (100% concentration) and water The wet weight and dry weight are the average of three repeated experiments. Test regularly once a day for seven consecutive days to count the results of dry and wet weight.

10.7717/peerj.11081/supp-16 Supplemental Information 16 The mRNA expression levels of asr in *E. coli* cells treated by different concentrations of *C. camphora* oil. *n* = 3 for each group

10.7717/peerj.11081/supp-17 Supplemental Information 17 The mRNA expression levels of dadX in *E. coli* cells treated by different concentrations of *C. camphora* oil. *n* = 3 for each group

10.7717/peerj.11081/supp-18 Supplemental Information 18 The mRNA expression levels of dadA in *E. coli* cells treated by different concentrations of *C. camphora* oil. *n* = 3 for each group

10.7717/peerj.11081/supp-19 Supplemental Information 19 The mRNA expression levels of HdeD in *E. coli* cells treated by different concentrations of *C. camphora* oil. *n* = 3 for each group

10.7717/peerj.11081/supp-20 Supplemental Information 20 The mRNA expression levels of Cph2 in *E. coli* cells treated by different concentrations of *C. camphora* oil. *n* = 3 for each group

10.7717/peerj.11081/supp-21 Supplemental Information 21 The mRNA expression levels of OmpC in *E. coli* cells treated by different concentrations of *C. camphora* oil. *n* = 3 for each group

10.7717/peerj.11081/supp-22 Supplemental Information 22 The mRNA expression levels of LamB in *E. coli* cells treated by different concentrations of *C. camphora* oil. *n* = 3 for each group

10.7717/peerj.11081/supp-23 Supplemental Information 23 The mRNA expression levels of PurD in *E. coli* cells treated by different concentrations of *C. camphora* oil. *n* = 3 for each group

10.7717/peerj.11081/supp-24 Supplemental Information 24 The mRNA expression levels of CsgD in *E. coli* cells treated by different concentrations of *C. camphora* oil. *n* = 3 for each group

10.7717/peerj.11081/supp-25 Supplemental Information 25 The mRNA expression levels of CysK in *E. coli* cells treated by different concentrations of *C. camphora* oil. *n* = 3 for each group

10.7717/peerj.11081/supp-26 Supplemental Information 26 The mRNA expression levels of RstA in *E. coli* cells treated by different concentrations of *C. camphora* oil. *n* = 3 for each group

10.7717/peerj.11081/supp-27 Supplemental Information 27 The mRNA expression levels of PurF in *E. coli* cells treated by different concentrations of *C. camphora* oil. *n* = 3 for each group

10.7717/peerj.11081/supp-28 Supplemental Information 28 The mRNA expression levels of ZraP in *E. coli* cells treated by different concentrations of *C. camphora* oil. *n* = 3 for each group

10.7717/peerj.11081/supp-29 Supplemental Information 29 The mRNA expression levels of ZraS in *E. coli* cells treated by different concentrations of *C. camphora* oil. *n* = 3 for each group

10.7717/peerj.11081/supp-30 Supplemental Information 30 The mRNA expression levels of raiA in *E. coli* cells treated by different concentrations of *C. camphora* oil. *n* = 3 for each group

10.7717/peerj.11081/supp-31 Supplemental Information 31 The mRNA expression levels of PurL in *E. coli* cells treated by different concentrations of *C. camphora* oil. *n* = 3 for each group

10.7717/peerj.11081/supp-32 Supplemental Information 32 The mRNA expression levels of evgs in *E. coli* cells treated by different concentrations of *C. camphora* oil. *n* = 3 for each group

## Additional Information and Declarations

### DNA Deposition

The following information was supplied regarding the deposition of DNA sequences: The RNA sequences described here are available at GEO: GSE160284.

### Data Availability

The following information was supplied regarding data availability: Raw data is available in the Supplemental Files.
