# Supplementary figures and images for: RNA-seq analysis of antibacterial mechanism of Cinnamomum camphora essential oil against Escherichia coli
Source: PeerJ. 2021 Mar 17;9:e11081. doi: 10.7717/peerj.11081 (PMC7980702; doi:10.7717/peerj.11081)

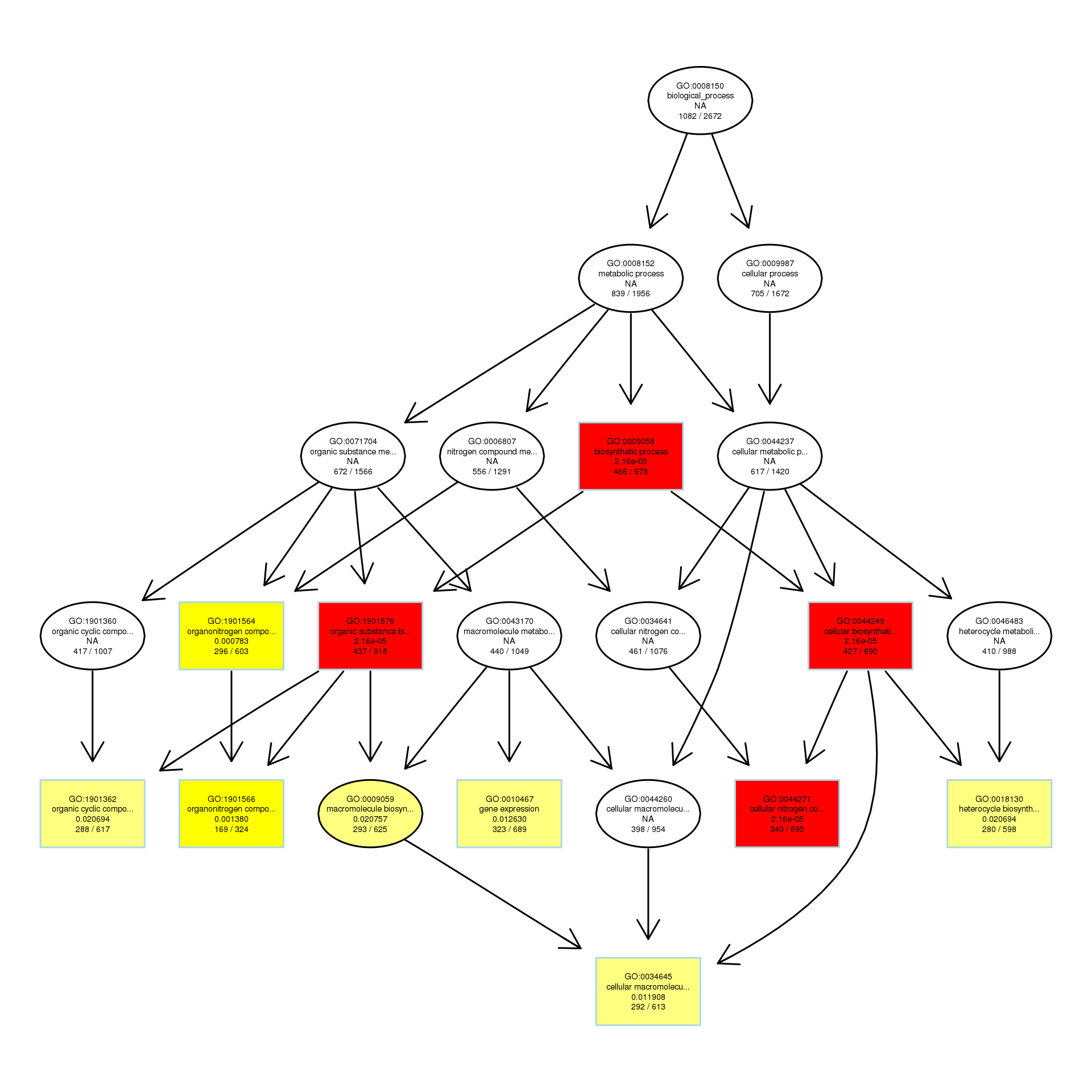

Supplement: Supplemental Information 1 — The DAG of BP in the down-regulated gene GO term. [file peerj-09-11081-s001.png]

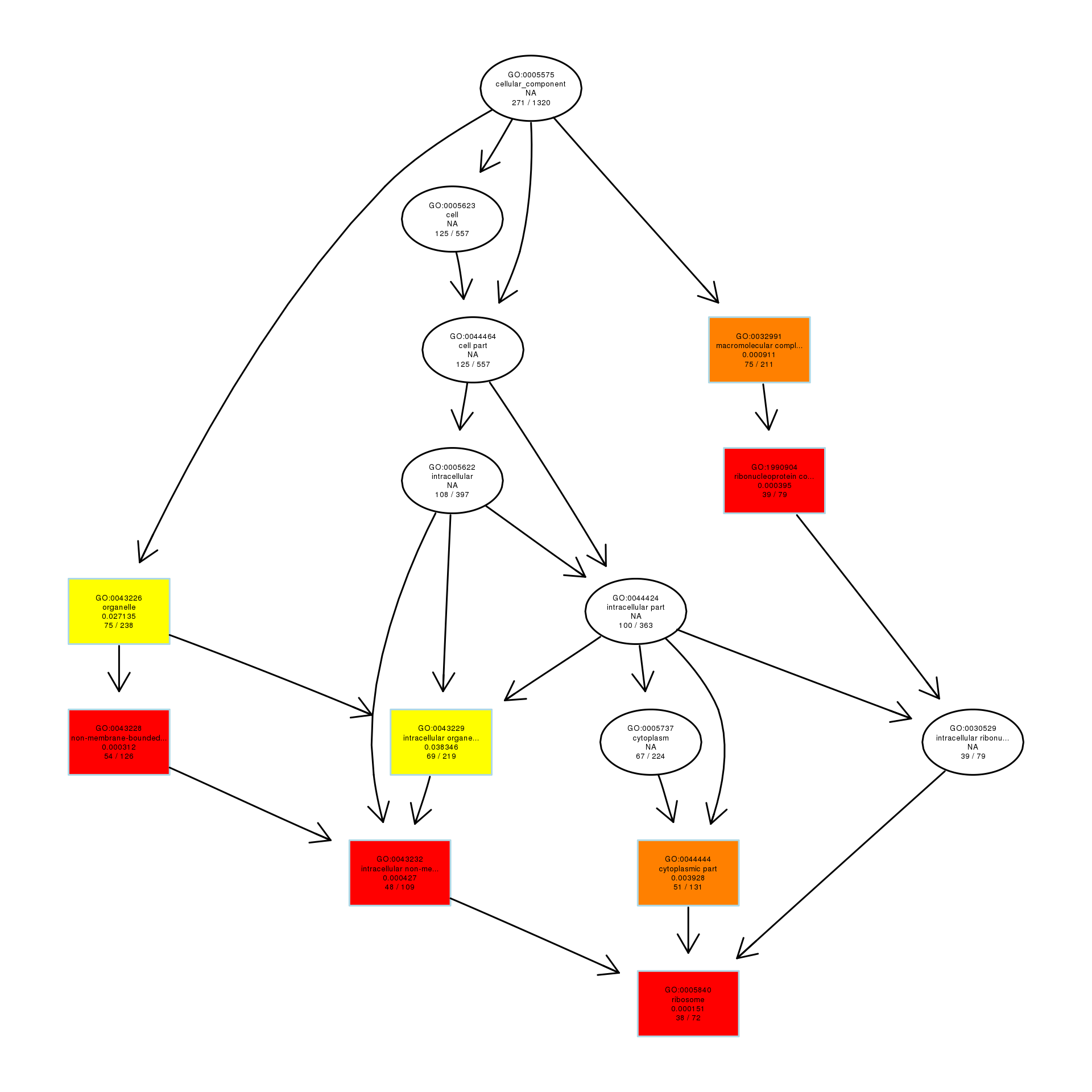

Supplement: Supplemental Information 2 — The DAG of CC in the down-regulated gene GO term. [file peerj-09-11081-s002.png]

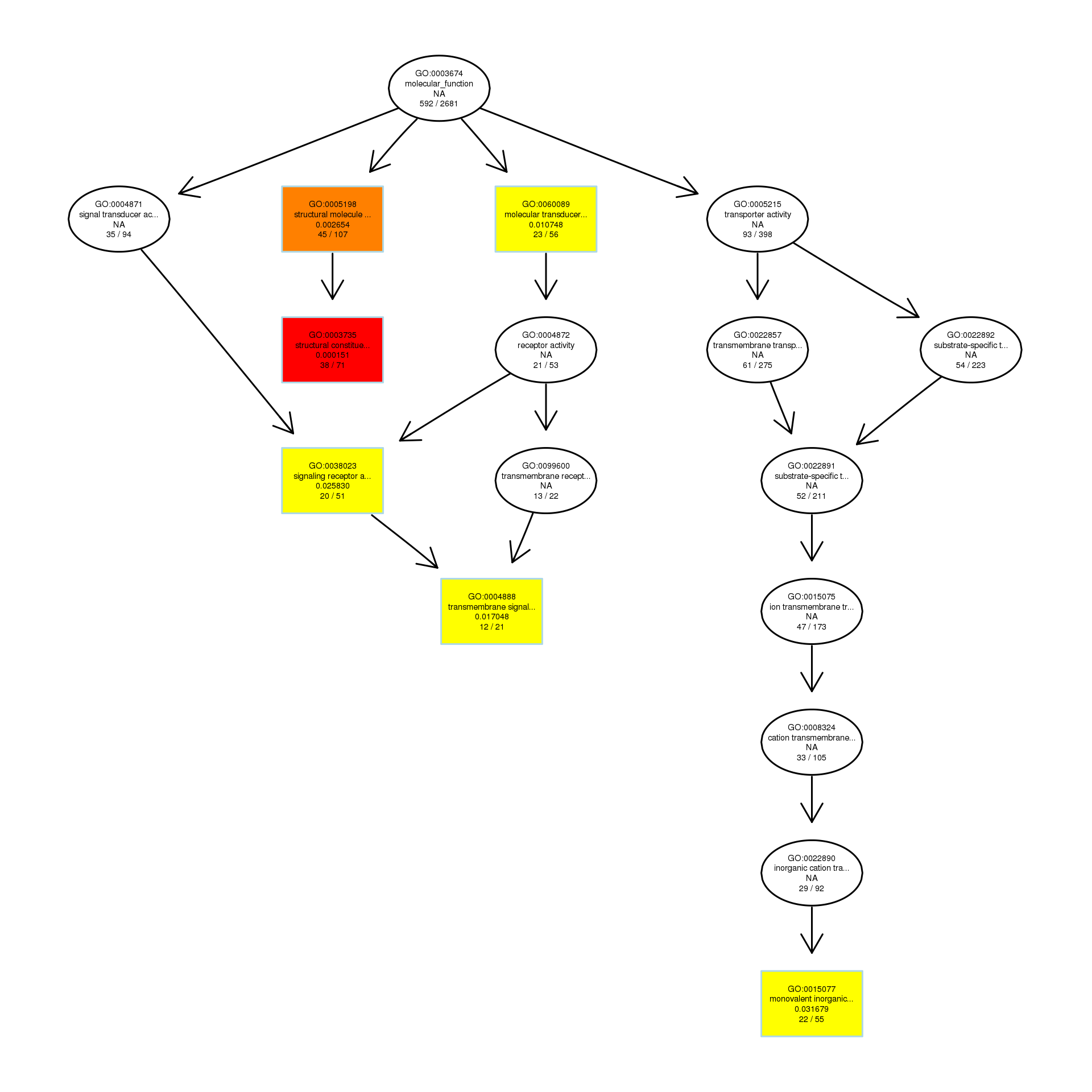

Supplement: Supplemental Information 3 — The DAG of MF in the down-regulated gene GO term. [file peerj-09-11081-s003.png]

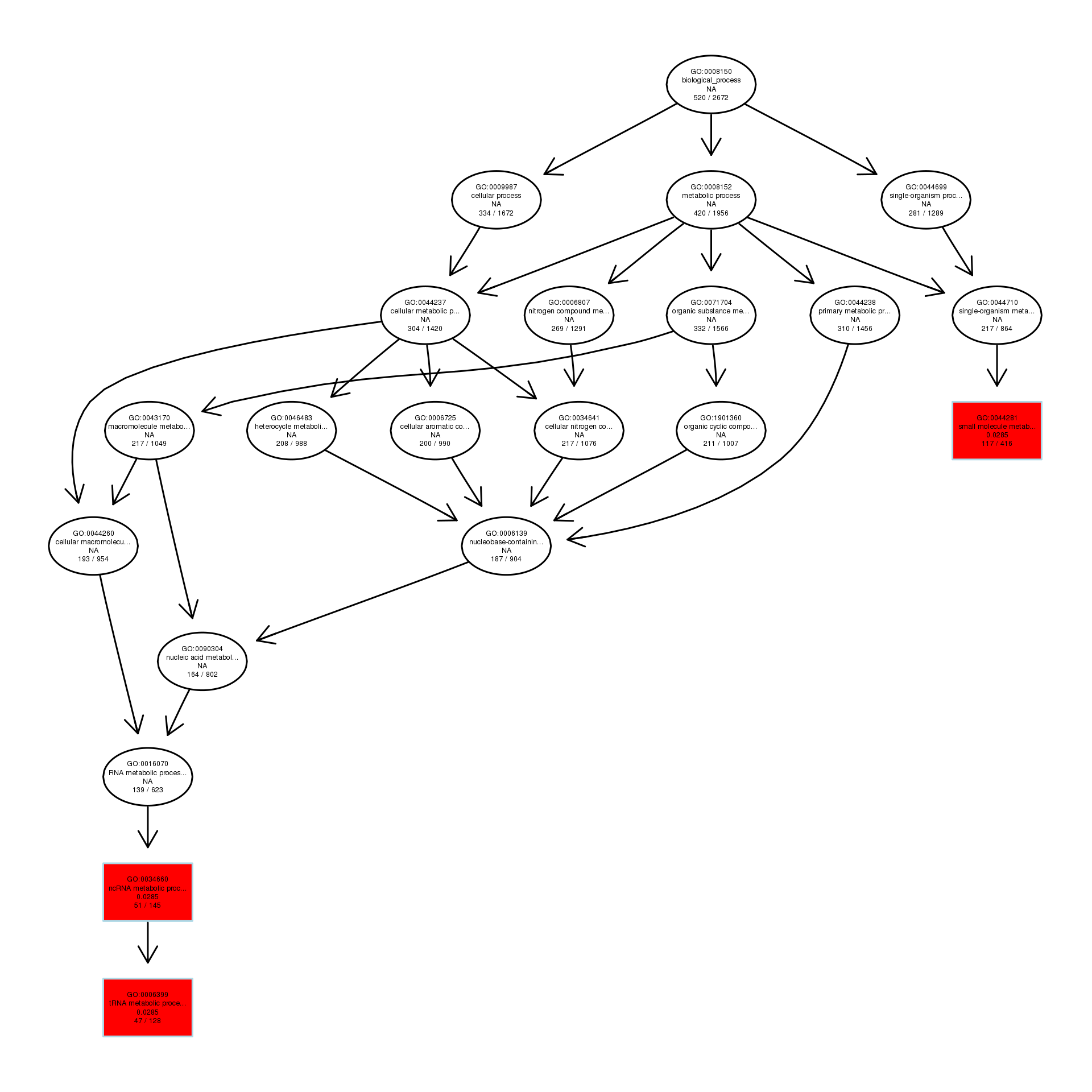

Supplement: Supplemental Information 4 — The DAG of BP in the up-regulated gene GO term. [file peerj-09-11081-s004.png]

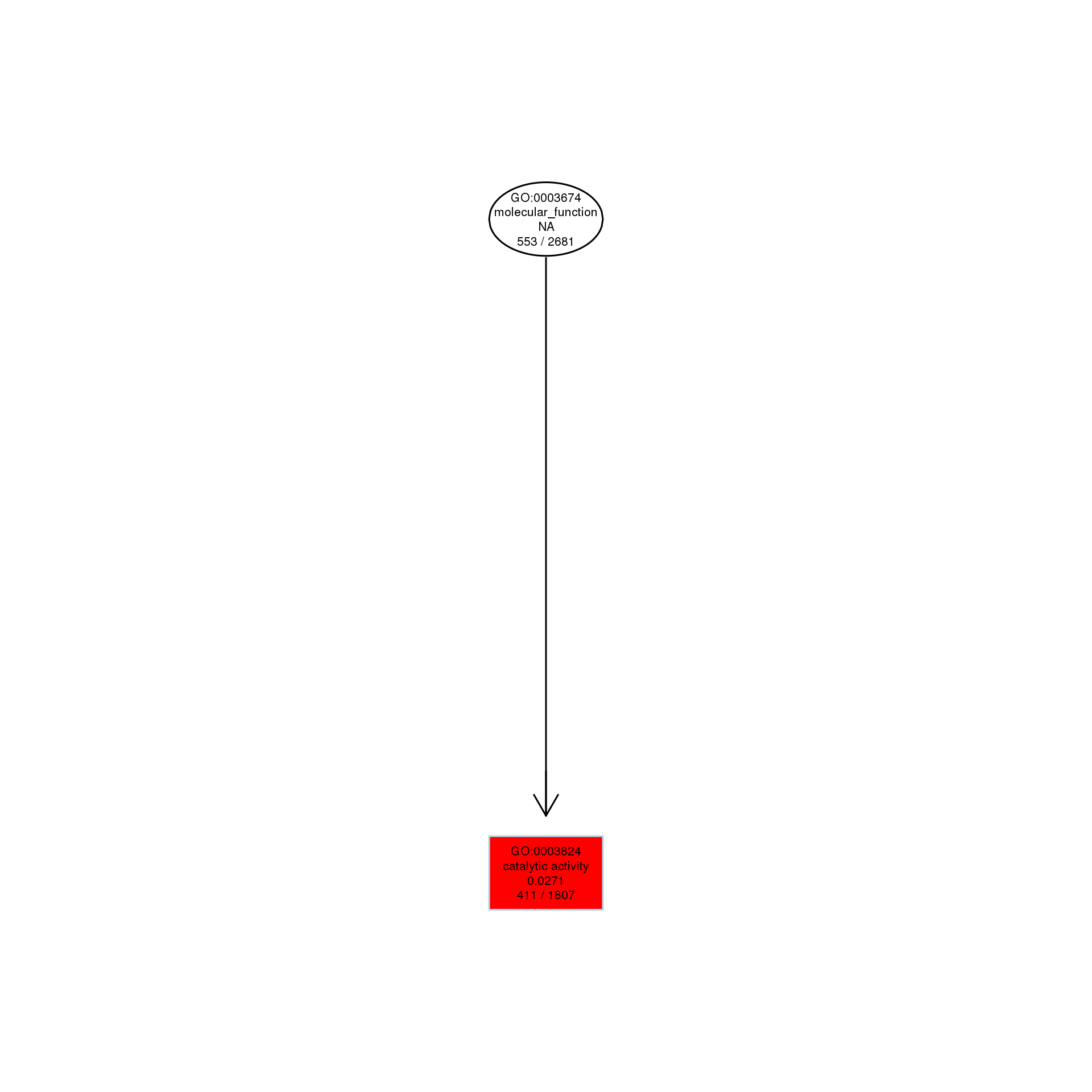

Supplement: Supplemental Information 5 — The DAG of MF in the up-regulated gene GO term. [file peerj-09-11081-s005.png]

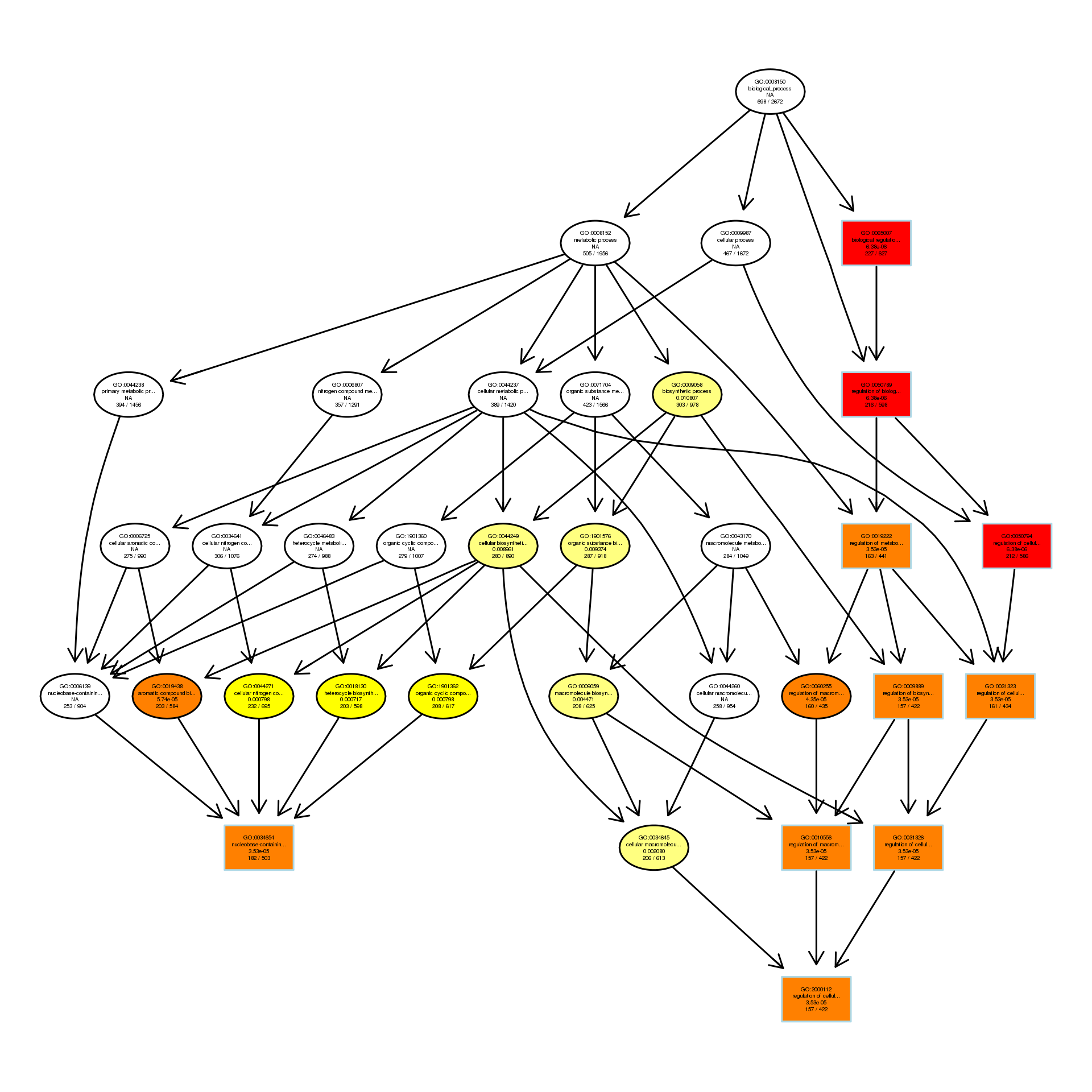

Supplement: Supplemental Information 6 — The DAG of BP in the down-regulated gene GO term. [file peerj-09-11081-s006.png]

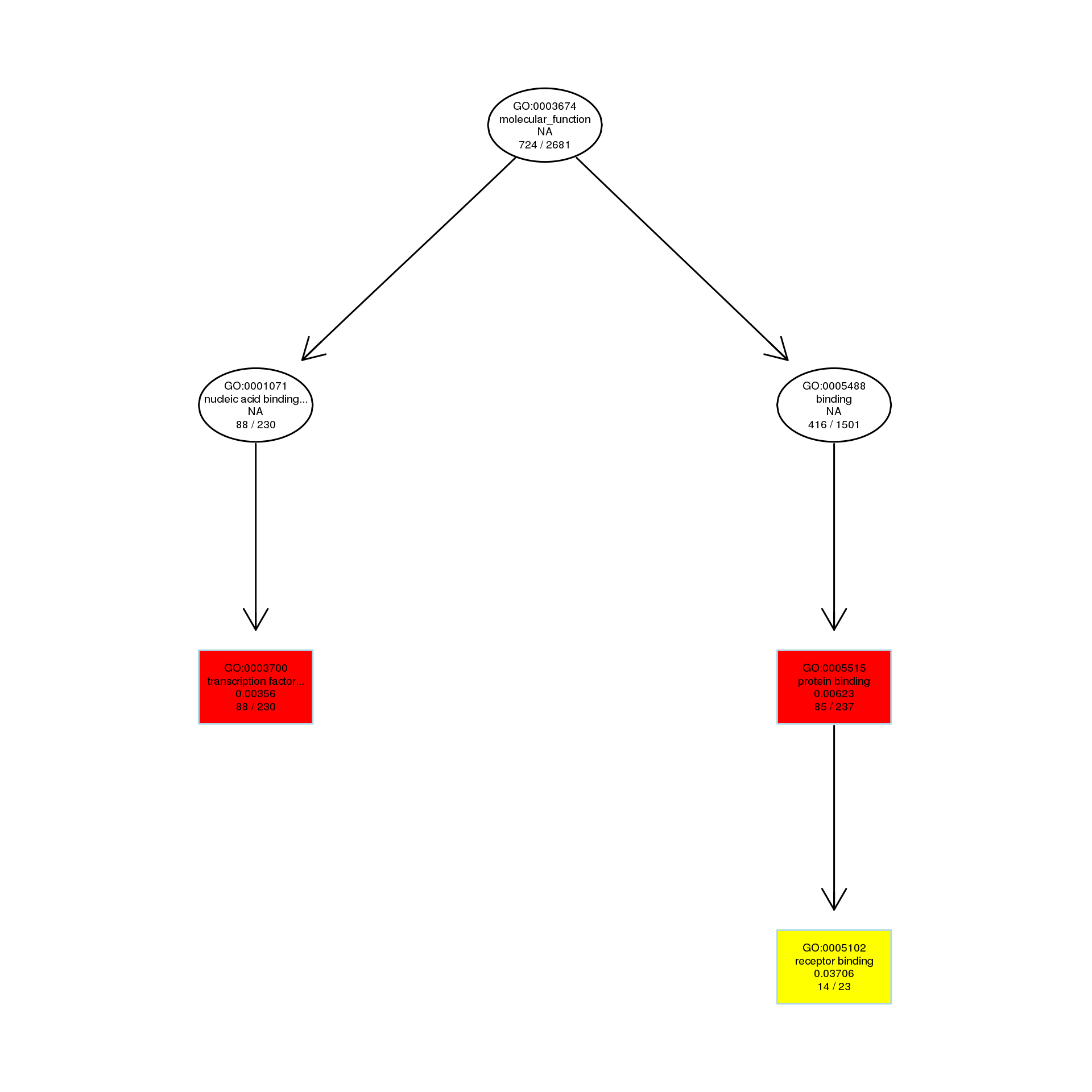

Supplement: Supplemental Information 7 — The DAG of MF in the down-regulated gene GO term. [file peerj-09-11081-s007.png]

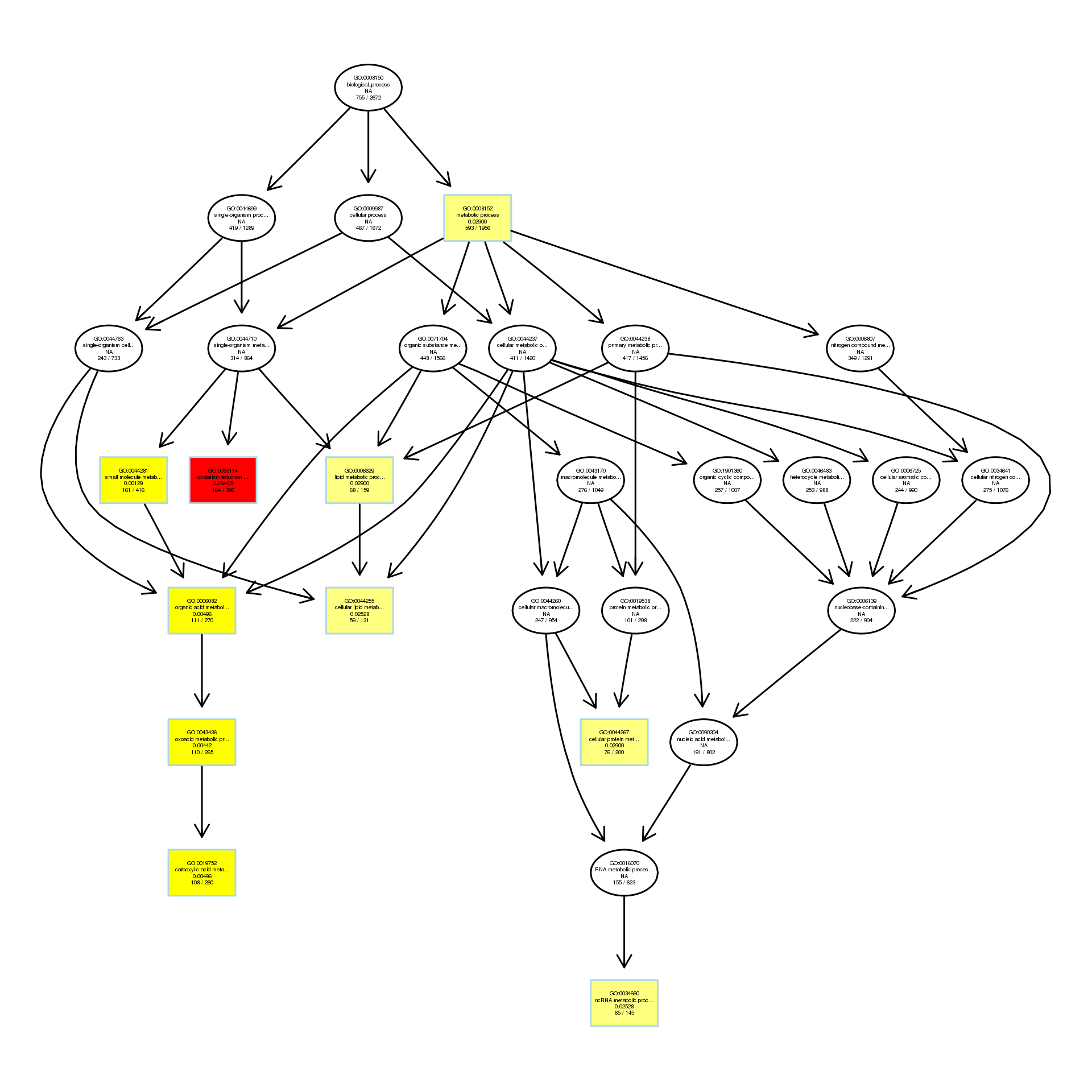

Supplement: Supplemental Information 8 — The DAG of BP in the up-regulated gene GO term. [file peerj-09-11081-s008.png]

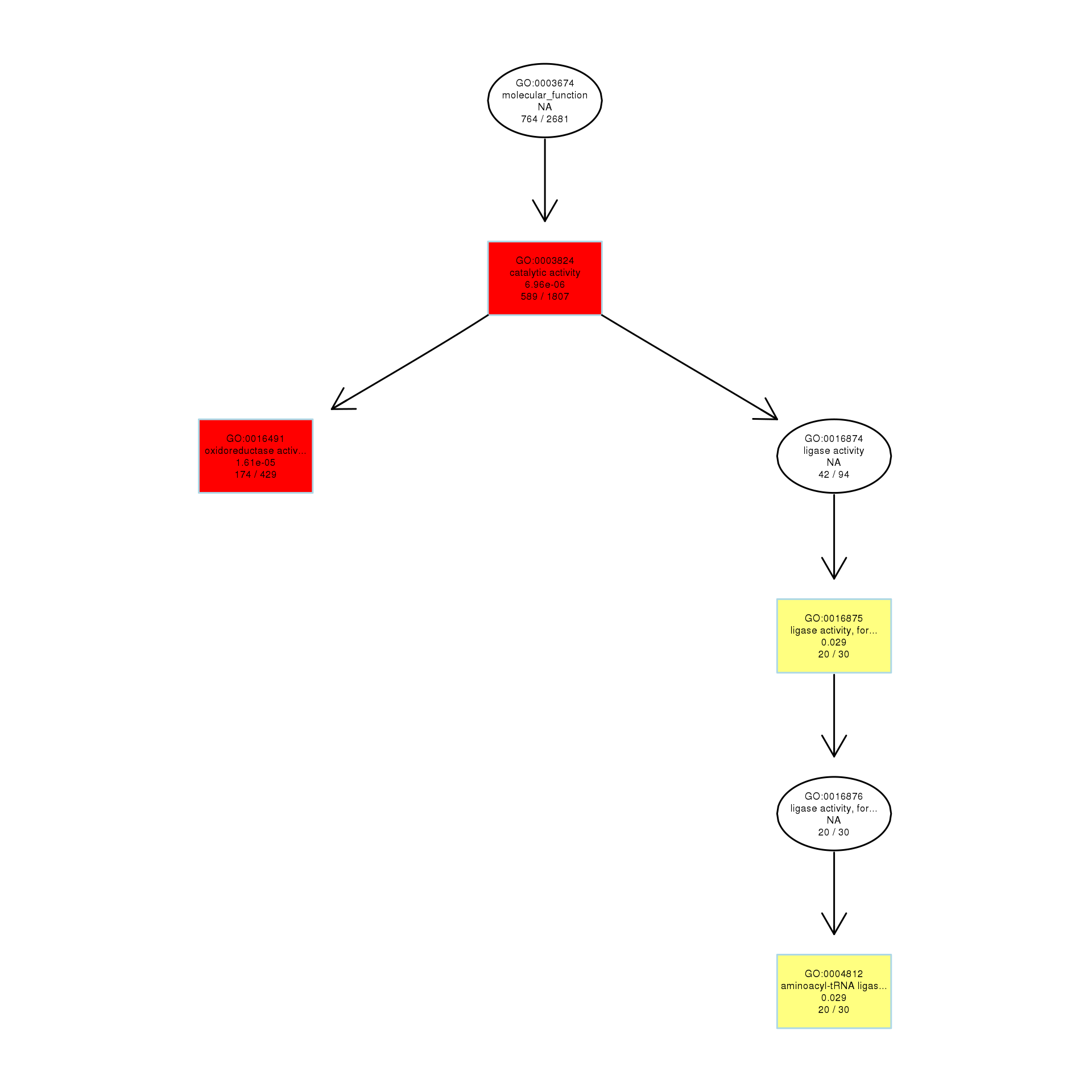

Supplement: Supplemental Information 9 — The DAG of MF in the up-regulated gene GO term. [file peerj-09-11081-s009.png]

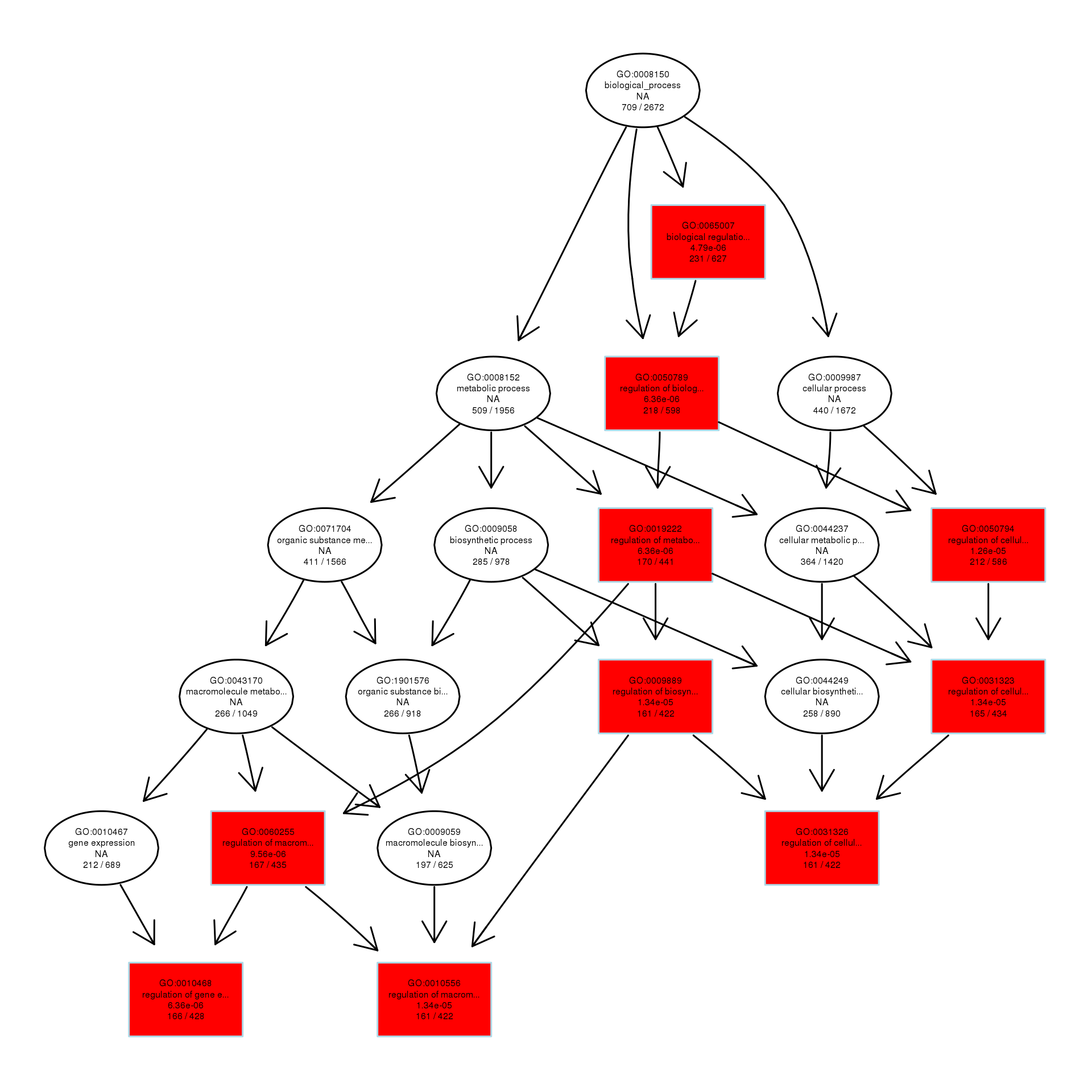

Supplement: Supplemental Information 10 — The DAG of BP in the down-regulated gene GO term. [file peerj-09-11081-s010.png]

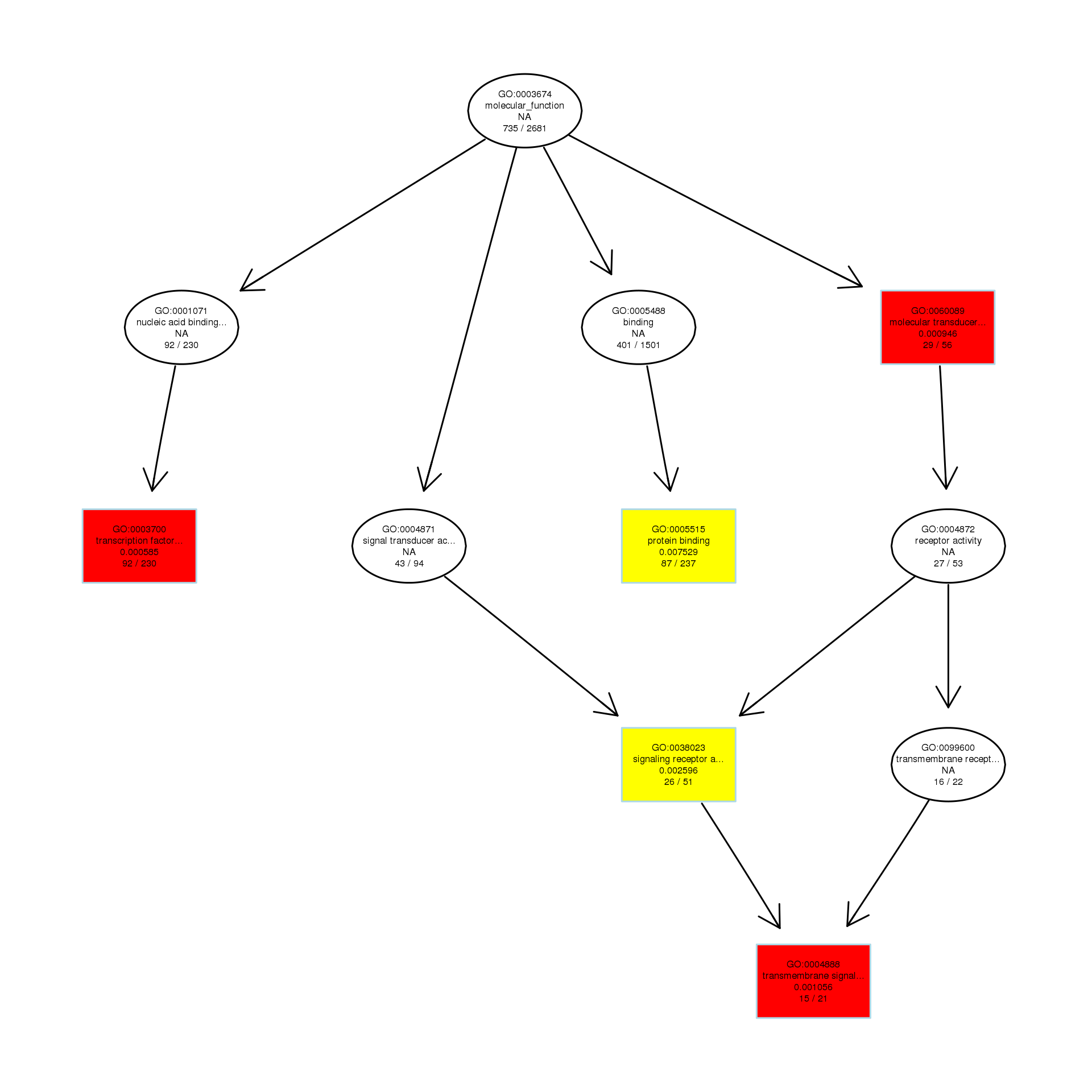

Supplement: Supplemental Information 11 — The DAG of MF in the down-regulated gene GO term. [file peerj-09-11081-s011.png]

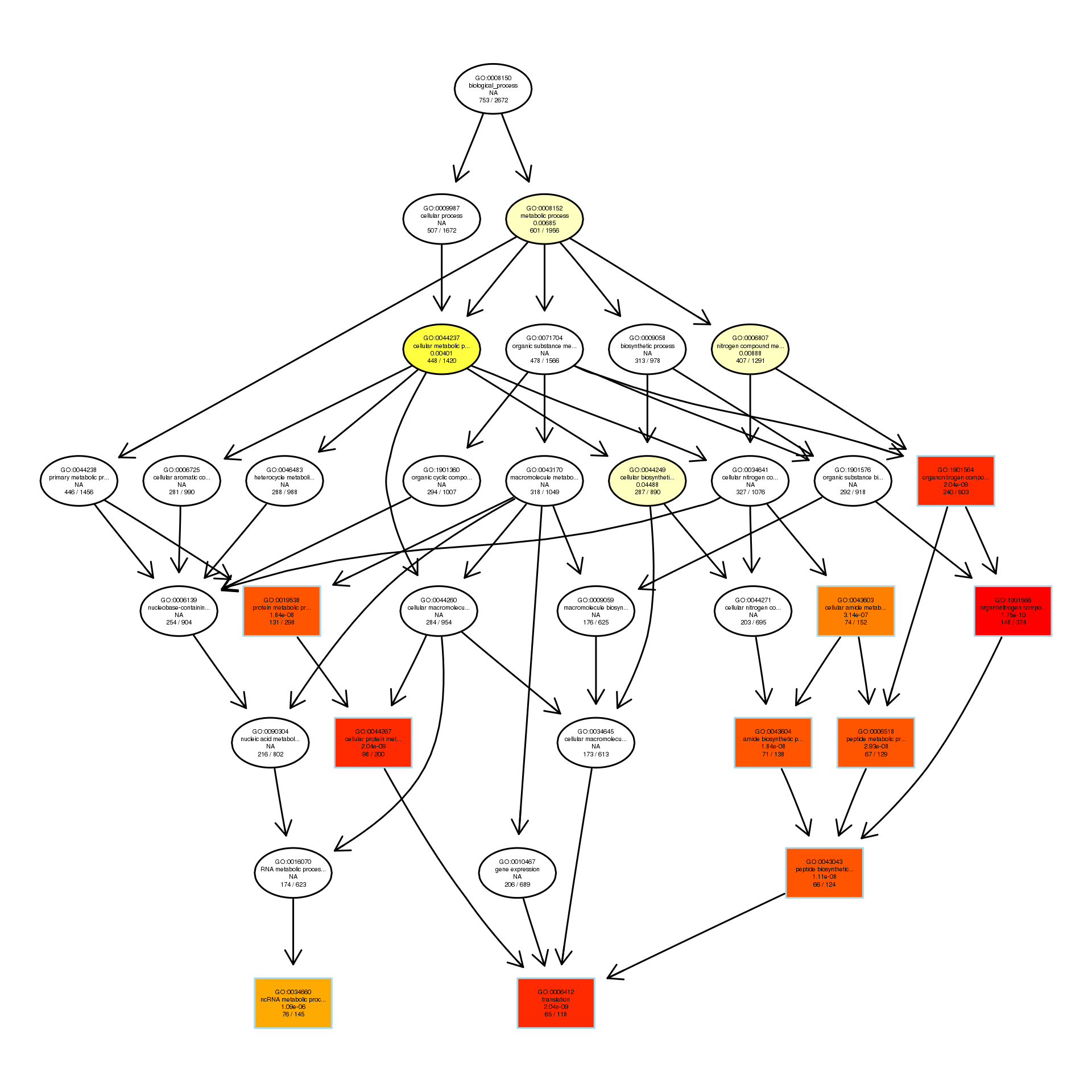

Supplement: Supplemental Information 12 — The DAG of BP in the up-regulated gene GO term. [file peerj-09-11081-s012.png]

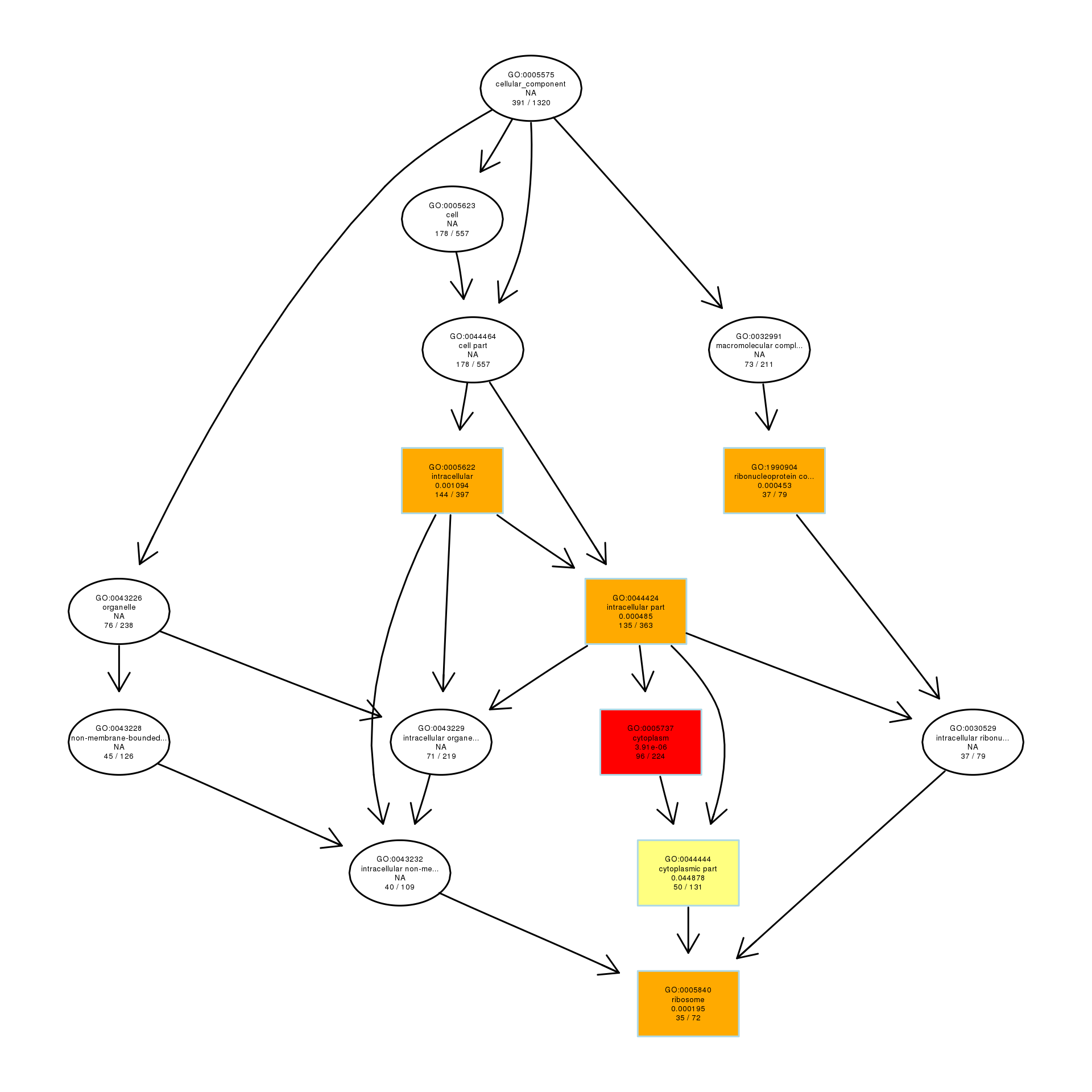

Supplement: Supplemental Information 13 — The DAG of CC in the up-regulated gene GO term. [file peerj-09-11081-s013.png]

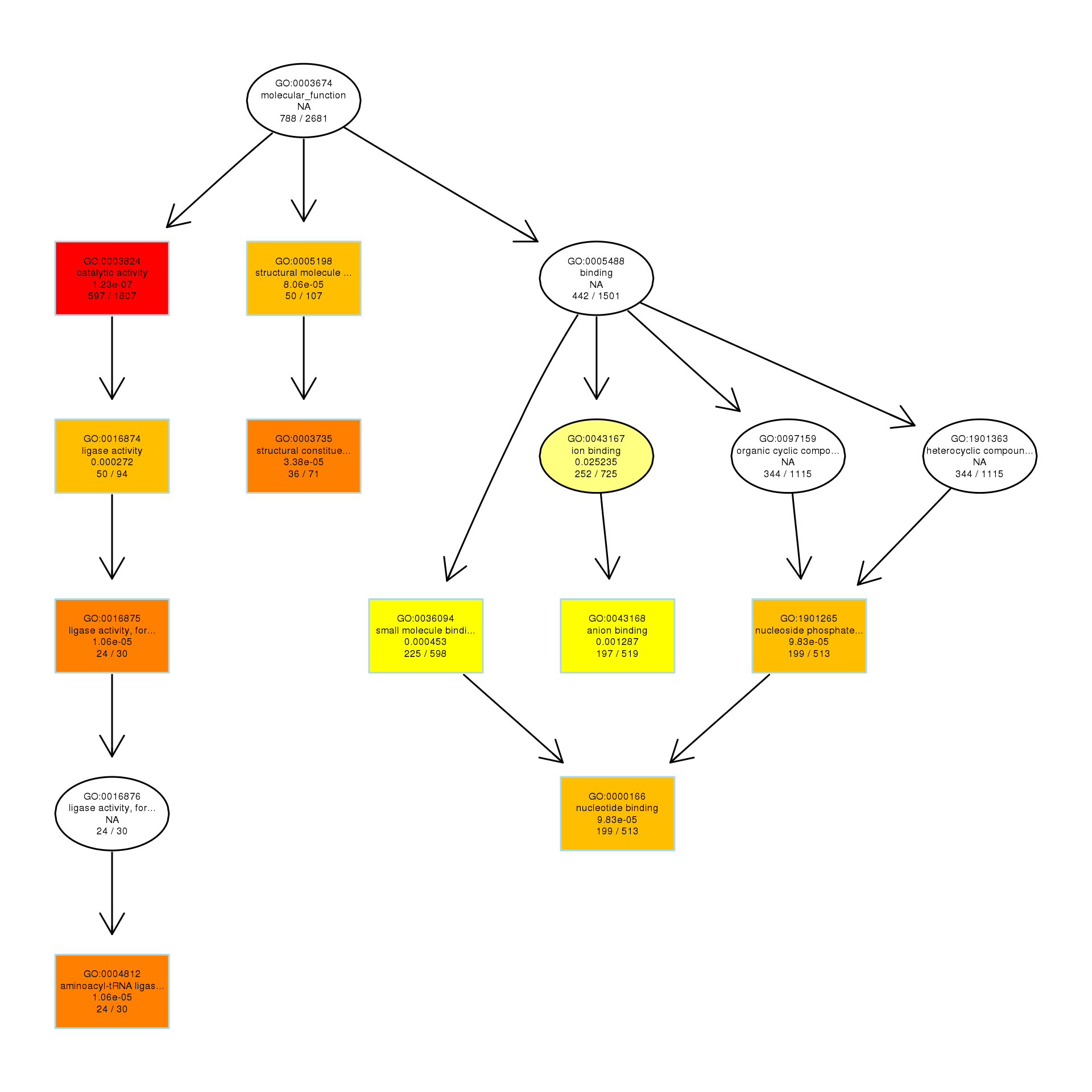

Supplement: Supplemental Information 14 — The DAG of MF in the up-regulated gene GO term. [file peerj-09-11081-s014.png]
